# Coexistence of mycobacterial infections – *Mycobacterium tuberculosis* and *Mycobacterium leprae* – in Sri Lanka: a case series

**DOI:** 10.1186/s13256-020-02413-w

**Published:** 2020-07-16

**Authors:** B. S. D. P. Keragala, H. M. M. T. B. Herath, G. H. D. C. Janapriya, S. Vanitha, Thanushah Balendran, Thavarajah Janani, T. S. Keragala, C. N. Gunasekera

**Affiliations:** 1 Dermatology Unit, National Hospital of Sri Lanka, Colombo, Sri Lanka; 2 Institute of Neurology, National Hospital of Sri Lanka, Colombo, Sri Lanka; 3 National Hospital of Sri Lanka, Colombo, Sri Lanka

**Keywords:** Tuberculosis, Leprosy, Coinfection, Case series

## Abstract

**Background:**

Leprosy is one of the oldest mycobacterial infections and tuberculosis is the most common mycobacterial infection with a higher degree of infectivity than leprosy. Although both diseases are prevalent in clusters in developing countries, simultaneous occurrence of them in an individual is a rare entity, even in an endemic setting.

**Case presentation:**

We describe six cases of tuberculosis and leprosy coinfection: a 57-year-old Sinhalese woman, a 47-year-old Tamil woman, a 72-year-old Tamil man, a 59-year-old Sinhalese man, a 54-year-old Sinhalese man, and a 50-year-old Sinhalese man. In this case series, five patients had lepromatous leprosy and the majority of patients were men. Three patients were detected to have tuberculosis at the outset of treatment of leprosy, while two developed tuberculosis later and one had extrapulmonary tuberculosis 5 years before the diagnosis of leprosy. The latter developed pulmonary tuberculosis as a reactivation while on treatment for leprosy. A majority of our patients with pulmonary tuberculosis had positive Mantoux test, high erythrocyte sedimentation rate, radiological evidence, and acid-fast bacilli in sputum. Human immunodeficiency virus and diabetes were detected in one patient. One patient had rifampicin-resistant tuberculosis, while she was on monthly rifampicin therapy for leprosy.

**Conclusion:**

An immunocompromised status, such as human immunodeficiency virus infection, diabetes, and immunosuppressive drugs, are risk factors for tuberculosis infection. The use of steroids in the treatment of leprosy may increase the susceptibility to develop tuberculosis. Development of rifampicin resistance secondary to monthly rifampicin in leprosy is a major concern in treating patients coinfected with tuberculosis. Despite the paucity of reports of coinfection, it is advisable to screen for tuberculosis in patients with leprosy, especially if there are respiratory or constitutional symptoms, high erythrocyte sedimentation rate, and abnormal chest X-ray. The fact is that positive Mantoux and QuantiFERON Gold tests and presence of acid-fast bacilli in sputum are misleading, chest X-ray evidence of active tuberculosis and positive tuberculosis cultures are important diagnostic clues for active tuberculosis infection in a patient with leprosy. This is important to avoid monthly rifampicin in patients with suspected coinfections, which may lead to development of drug resistance to tuberculosis treatment. Whether prolonged steroid therapy in leprosy is a risk factor for development of tuberculosis is still controversial.

## Introduction

Tuberculosis (TB) is a multisystem disease and one of the most common infectious causes of deaths worldwide. Leprosy is an infectious disease that involves the skin and peripheral nerves. Both TB and leprosy are granulomatous infections caused by the intracellular Gram-positive aerobic acid-fast bacilli (AFB) *Mycobacterium tuberculosis* and *Mycobacterium leprae*, respectively. Clinical manifestations of both diseases vary according to the host’s immune status and response. TB, at one pole, can present as miliary TB, which is anergic and multibacillary and, at the other pole, as lupus vulgaris and verrucous TB, which are hyperergic and paucibacillary, while erythema induratum of Bazin is an immune-mediated hypersensitivity reaction for TB [1]. In leprosy, at one pole, tuberculoid leprosy represents a paucibacillary form and, at the other pole, lepromatous leprosy represents the multibacillary form [2]. Immunological reactions to leprosy manifest as lepra reactions that can occur before, during, or even years after completion of treatment.

Both are prevalent in clusters in developing countries; however, the simultaneous occurrence of both infections in an individual is rare even in endemic areas (0.02 per 100,000 population) [3]. Only a few case reports of the coexistence of TB and leprosy in the same patients are available in the literature. In this case series we describe six Sri Lankan patients who were infected with both TB and leprosy over a period of 7 years starting from 2012 and we briefly review the literature of coinfection.

## Case presentations

### Case 1

**Fig. 1:**
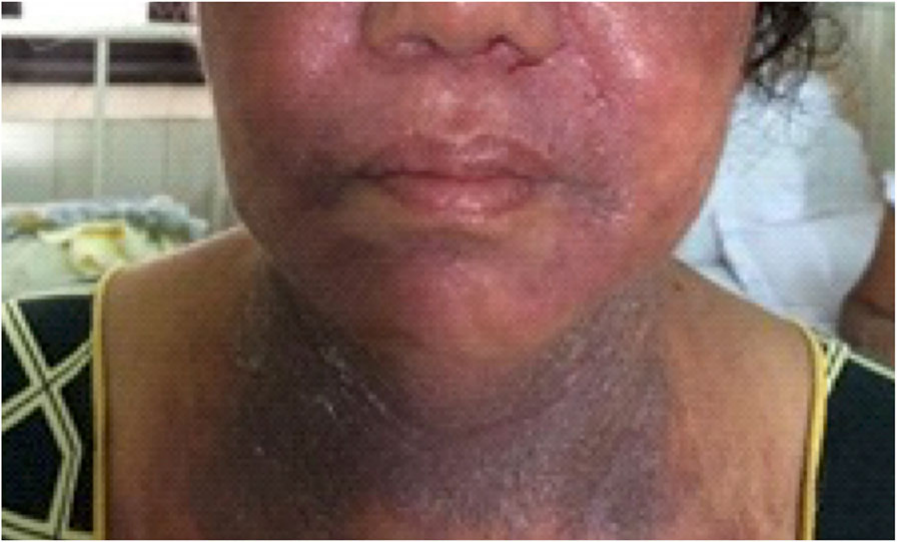
Extensive seborrhoeic dermatitis and tender facial induration

A 57-year-old Sinhalese woman presented to the dermatology unit of the National Hospital of Sri Lanka with extensive seborrheic dermatitis and tender facial induration (Fig. 1) of 1-year duration. She was a homemaker from an urban area with poor social background. She did not have diabetes or hypertension. She had received a Bacillus Calmette–Guérin (BCG) vaccination. She did not smoke tobacco or drink alcohol. A skin biopsy taken from indurated facial plaque revealed evidence of borderline tuberculoid leprosy with type 1 reaction. She did not have hypopigmented anesthetic patches or thickened peripheral nerves in the rest of her body. She was commenced on multibacillary treatment for leprosy. A year later, she developed fever, loss of appetite, loss of weight, and widespread skin abscesses all over her body. Her body mass index (BMI) was within normal range and the rest of the general examination was normal. A respiratory examination revealed bilateral (B/L) coarse crepitations; a neurological examination was normal. Her erythrocyte sedimentation rate (ESR) remained persistently elevated at more than 120 mm/hour. She had inflammatory shadows in a chest X-ray (CXR). A sputum examination for AFB was positive and GeneXpert confirmed rifampicin resistance. At that time, she revealed a diagnosis of human immunodeficiency virus (HIV) infection and tuberculous lymphadenitis, which was made 5 years back, where she had taken 6-month TB treatment but had defaulted on antiretroviral therapy. During this presentation, her CD4+ count was 57/μL. The rest of the investigations are summarized in Table 1. There was no contact history of leprosy or TB. She was started on a multidrug treatment regime, which included intravenously administered kanamycin (15 mg/kg per day), orally administered ethambutol (800 mg/day), orally administered levofloxacin (750 mg/day), isoniazid (300 mg/day), orally administered ethionamide (500 mg/day), orally administered cycloserine (750 mg/day), and orally administered pyridoxine (10 mg/day). A repeated split skin smear was negative and she continued with multidrug treatment-multibacillary therapy (dapsone 100 mg/day and clofazimine 50 mg/day and 300 mg/once a month) for leprosy without rifampicin. Highly active antiretroviral treatment was restarted subsequently. At 6 months she had clinically, hematologically, and radiologically improved.

**Table 1:** Basic investigations of six cases of tuberculosis and leprosy coinfection

	WBC 10^3^/μL	Hb g/dL	PLT 10^3^/μL	SC mg/dL	Sodium mmol/L	AST U/L	ALT U/L	Alb g/L	Glob g/L	UFR
Case 1	3.3	10.6	150	1.13	141	45	50	2.6	3.0	Normal
Case 2	6.0	12.3	202	0.9	137	35	56	3.5	4.3	Normal
Case 3	8.0	13.9	222	1.4	138	33	45	3.4	3,5	Normal
Case 4	6.8	11.5	230	0.9	140	44	54	3.0	4.2	Normal
Case 5	5.7	13.2	190	1.1	135	60	70	3.5	2.9	Normal
Case 6	6.5	13.5	240	1.3	137	49	45	3.4	3.6	Normal

*Alb* albumin, *ALT* alanine transaminase, *AST* aspartate transaminase, *Glob* globulin, *Hb* hemoglobin, *PLT* platelet, *SC* serum creatinine, *UFR* urine full report, *WBC* white blood cells

### Case 2

**Fig. 2:**
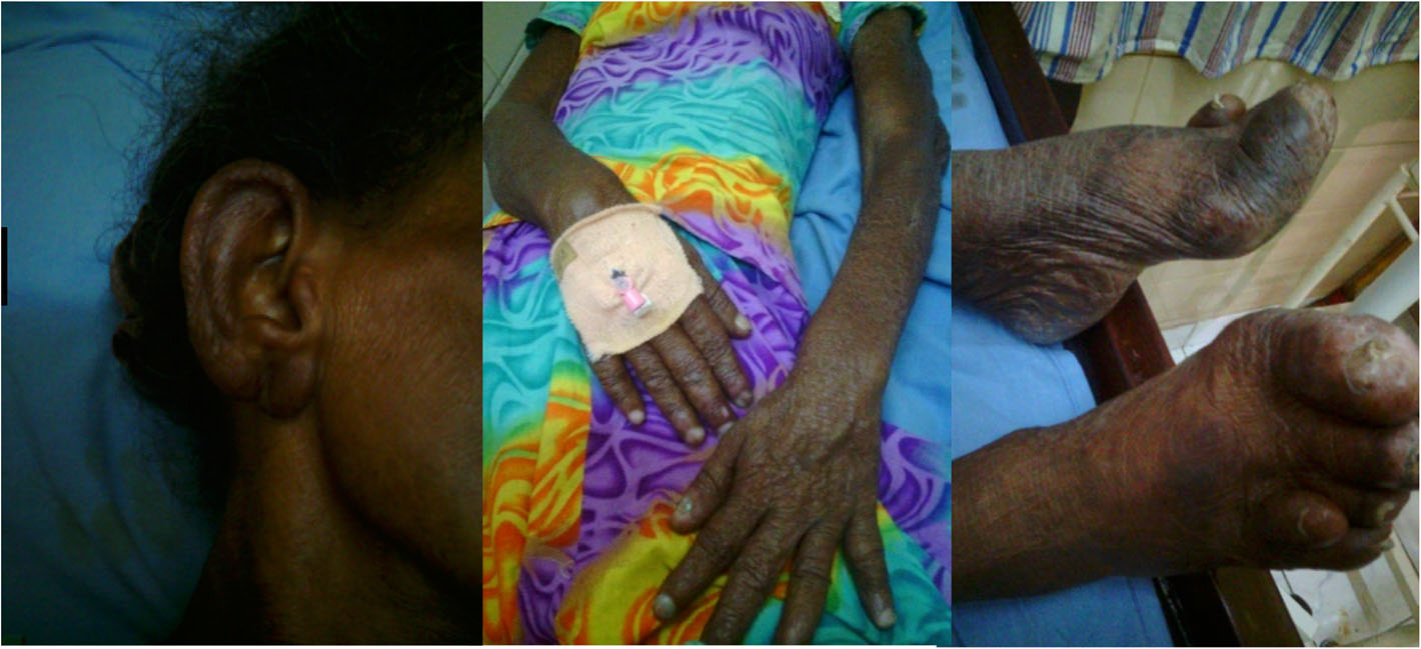
Facial infiltration, multiple ichthyotic patches, and peripheral neuropathy associated with Charcot joints and trophic ulcers

A 47-year-old Tamil woman with poorly controlled long-standing diabetes mellitus presented with facial infiltration, multiple ichthyotic patches, and peripheral neuropathy associated with Charcot joints and trophic ulcers (Fig. 2). She was a homemaker with poor social background and there was no contact history of TB or leprosy. She did not smoke tobacco or drink alcohol. She had received a BCG vaccination. She had a low BMI and a respiratory examination revealed B/L coarse crepitations. There was no peripheral neuropathy. A clinical diagnosis of lepromatous leprosy was made which was later confirmed with histology and slit-skin smears: bacteriological index (BI) of 5; morphological index (MI) of 2%. Initial investigations revealed persistently elevated ESR (100–122 mm/hour) and inflammatory shadows in a CXR. Two consecutive sputum smears were positive for AFB and the result of a Mantoux test was positive (18 mm). Her liver and renal functions were normal (Table 1). She was negative for HIV screening. She was commenced on both TB (isoniazid 300 mg/day, rifampicin 450 mg/day, ethambutol 800 mg/day, and pyrazinamide 1500 mg/day) and multibacillary leprosy treatment (dapsone 100 mg/day and clofazimine 50 mg/day and 300 mg/once a month) without a monthly loading dose of rifampicin. She had poor compliance and defaulted treatment several times and ultimately succumbed to the illness. An autopsy was not performed.

### Case 3

**Fig. 3:**
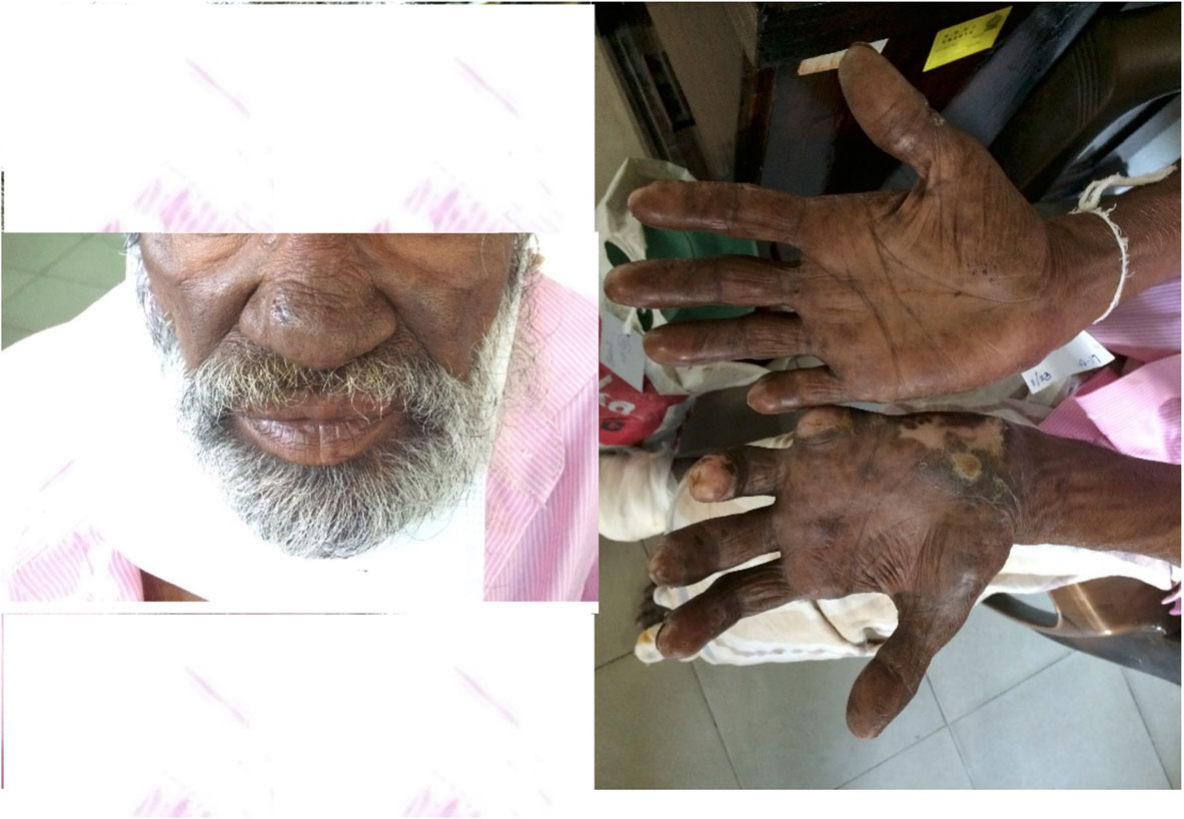
Trophic ulcers, auto-amputated fingers, and facial infiltration

A 72-year-old Tamil man presented with a 2-year history of trophic ulcers, auto-amputated fingers, and facial infiltration (Fig. 3). He was a retired mason from a poor social back ground with low income and did not have any other medical diseases. A general examination and neurological examination was normal. There was no significant contact history of leprosy or TB. He had received a BCG vaccination. He was an ex-smoker of tobacco and an ex-drinker of alcohol. His evaluation confirmed the clinical diagnosis of leprosy with highly positive slit-skin smears and histological evidence of lepromatous leprosy. He also gave a history of constitutional symptoms, evening pyrexia, and loss of weight and appetite. His ESR was 113 mm/hour and a sputum examination revealed AFB along with positive Mantoux test of 14 mm and CXR evidence of pulmonary TB. Basic investigations are given in Table 1. He was evaluated for both congenital and acquired immunodeficiency status, which did not reveal any abnormality. Although treatment for both conditions was commenced (isoniazid 300 mg/day, rifampicin 450 mg/day, ethambutol 800 mg/day, and pyrazinamide 1500 mg/day and dapsone 100 mg/day and clofazimine 50 mg/day and 300 mg/once a month) in liaison with a respiratory team, he was lost to follow up.

### Case 4

**Fig. 4:**
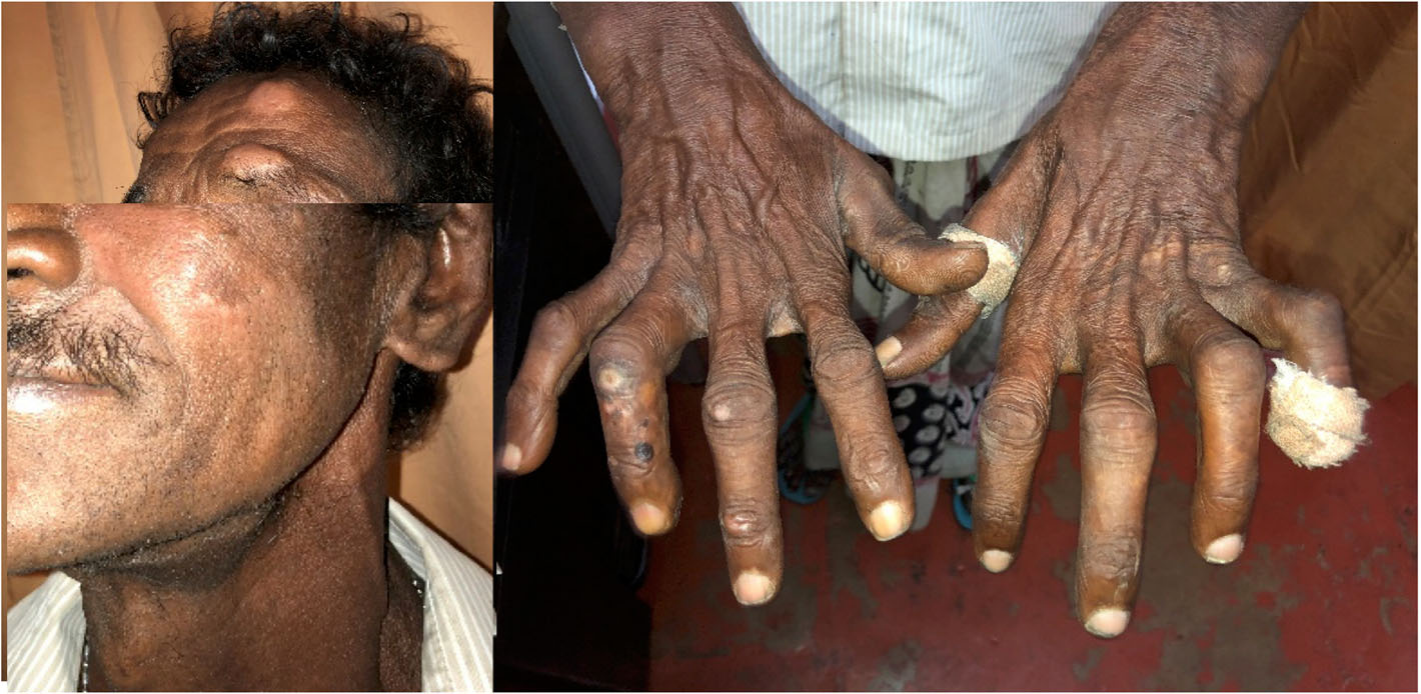
Nonhealing ulcers, deformities of both upper and lower limbs, and indurations of the face

A 59-year-old Sinhalese man presented with nonhealing ulcers and deformities of both upper and lower limbs and indurations of his face (Fig. 4) for many years. He was clinically diagnosed to have borderline lepromatous leprosy, which was subsequently confirmed with slit-skin smears and histology. He was a laborer from poor social background and did not have a significant past medical history. He was emaciated with low BMI; a neurological examination did not reveal neuropathy. There was no contact history of leprosy or TB. He had received a BCG vaccination. He did not smoke tobacco and he drank alcohol occasionally. During his initial evaluation he was also found to have high ESR (> 100 mm/hour) with positive Mantoux test (21 mm) and sputum was positive for AFB. A CXR and contrast-enhanced computer tomography scan of his chest revealed the focus of pulmonary TB; bronchoalveolar lavage for TB culture revealed growth of AFB, but GeneXpert did not reveal rifampicin resistance. His HIV status was negative and repeated blood sugar levels remained within normal range. Other basic investigations are given in Table 1. He was commenced on multibacillary leprosy treatment (dapsone 100 mg/day and clofazimine 50 mg/day and 300 mg/once a month) and subsequently added TB treatment (isoniazid 300 mg/day, rifampicin 450 mg/day, ethambutol 800 mg/day, and pyrazinamide 1500 mg/day) for 6 months. At 6 months, his ESR came down, sputum was negative for AFB, and skin lesions and ulcers improved.

### Case 5

**Fig. 5:**
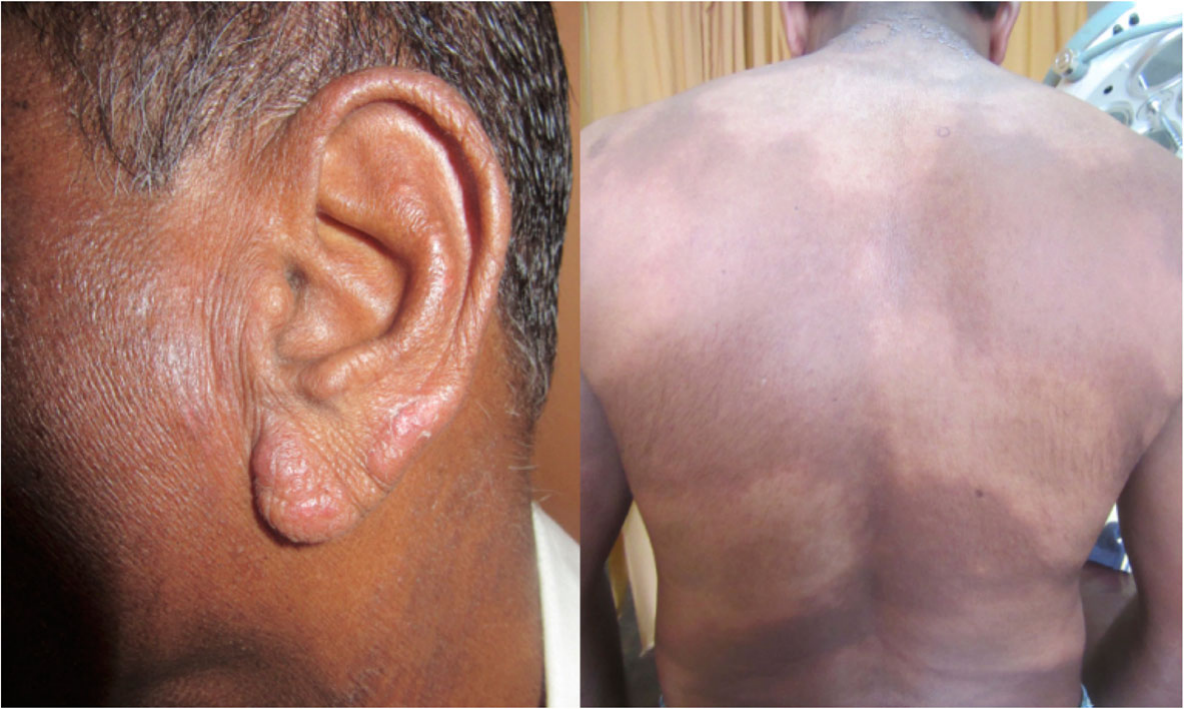
Asymptomatic infiltrated papules and plaques over the earlobes of 5 months’ duration and hypopigmented hypoanesthetic patches all over the body of similar duration

A 54-year-old Sinhalese man, an intravenous substance abuser, a tobacco smoker, and alcoholic, who was a garbage truck worker by occupation, with a close contact history of leprosy (his daughter), presented with leonine face and asymptomatic infiltrated papules and plaques over his earlobes of 5 months’ duration and hypopigmented hypoanesthetic patches all over his body for a similar duration (Fig. 5). His skin smear and skin biopsy were compatible with borderline lepromatous leprosy. He had a normal BMI; a neurological examination was normal. He had received a BCG vaccination. He was commenced on multibacillary treatment for leprosy (dapsone 100 mg/day, clofazimine 50 mg/day and 300 mg/once a month and rifampicin 600 mg/month) with tailing-off of dose of prednisolone starting from 40 mg daily. Six months later, he presented with productive cough with nocturnal pyrexia; an evaluation revealed high ESR (130 mm/hour), CXR evidence of active pulmonary TB, and positive sputum smear for AFB. Immune function studies carried out to evaluate his immunodeficiency status did not reveal any congenital or acquired immunodeficiency conditions, including diabetes and HIV. Table 1 gives the basic investigations. He was commenced on TB (isoniazid 300 mg/day, rifampicin 450 mg/day, ethambutol 800 mg/day, and pyrazinamide 1500 mg/day) treatment. He defaulted treatment several times with regards to both leprosy and TB and later died due to TB. An autopsy was not performed.

### Case 6

**Fig. 6:**
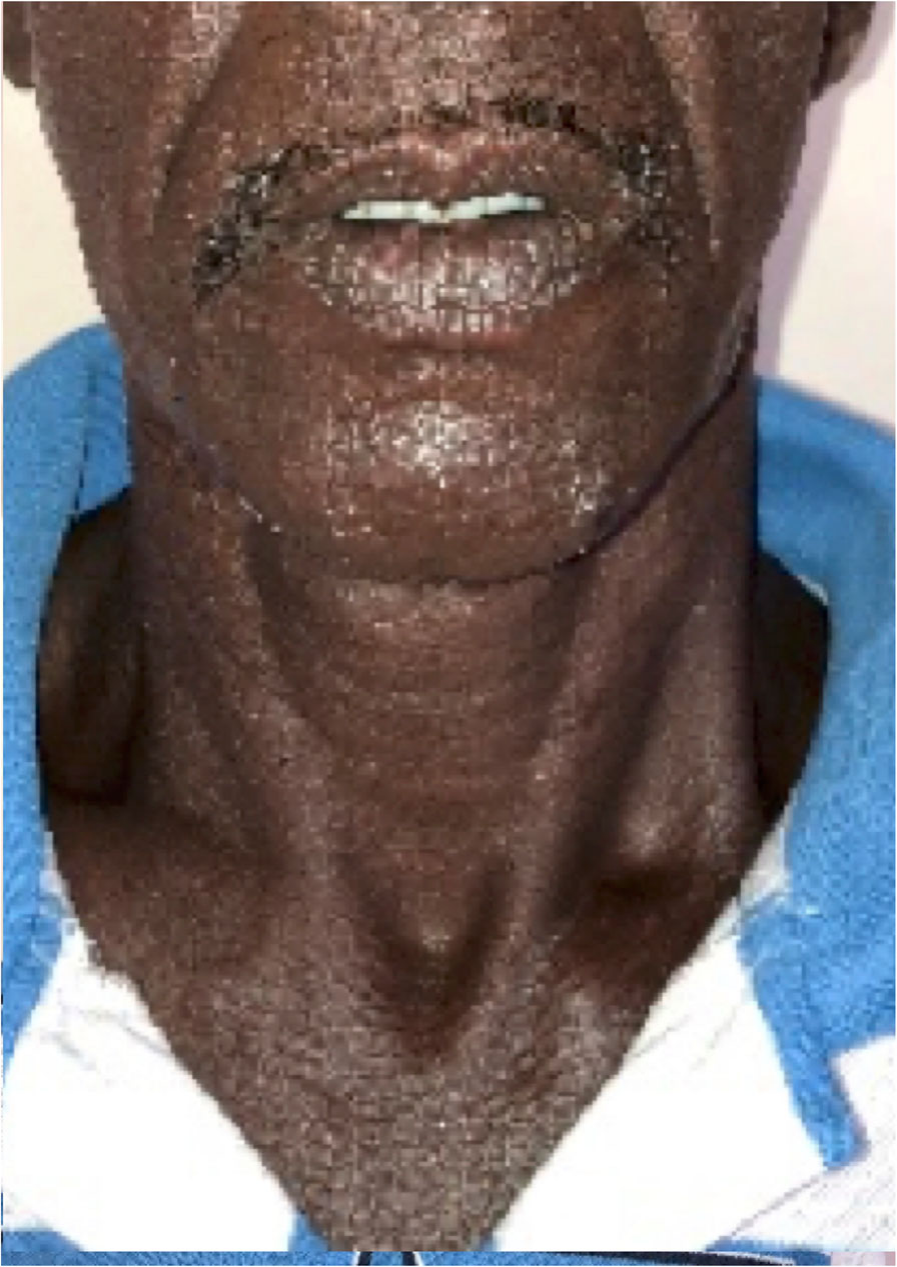
Facial infiltration

A 50-year-old Sinhalese man with poorly controlled diabetes mellitus presented with facial infiltration (Fig. 6) and erythematous painful nodules over his lower limbs and trunk suggestive of lepromatous leprosy with erythema nodosum leprosum. He was a manual laborer with low BMI. The rest of the general and neurological examination was normal. He had received a BCG vaccination. He did not smoke tobacco and drank alcohol occasionally. A skin biopsy revealed evidence of foamy macrophages with well-developed grenz zone. He was commenced on multidrug treatment-multibacillary treatment for leprosy (dapsone 100 mg/day, clofazimine 50 mg/day and 300 mg/once a month, and rifampicin 600 mg/month) with prednisolone 40 mg daily for type 2 reaction. Twelve months later he developed generalized lymphadenopathy with low-grade evening pyrexia and elevated ESR (80–100 mm/hour). Both Mantoux and QuantiFERON Gold test were positive. A CXR remained persistently normal. He was negative for HIV and diabetes. Basic investigations are given in Table 1. A lymph node excision biopsy revealed suppurative inflammation with focal foamy histiocytes; a TB culture from the lymph node biopsy later showed growth of AFB. GeneXpert did not reveal rifampicin resistance. Therefore, he was started on TB treatment (isoniazid 300 mg/day, rifampicin 450 mg/day, ethambutol 800 mg/day, and pyrazinamide 1500 mg/day) by the respiratory physician for the treatment of extrapulmonary TB. At 6 months, he had improved clinically and biochemically.

## Discussion

Here we describe six cases of TB and leprosy coinfection (Table 2). TB infection is seen across the entire spectrum of leprosy [4, 5]. In our case series, three patients had lepromatous leprosy, two patients had borderline tuberculoid leprosy, and one had borderline lepromatous leprosy. Four patients were men and two were women. These findings are similar to the case series of Nigam and colleagues, where out of 20 cases, the majority had lepromatous leprosy and a majority were men [5]. In addition, in the literature, most of the coinfections of TB with leprosy were cases of lepromatous leprosy followed by borderline lepromatous leprosy and only a few cases of tuberculoid type of leprosy [6, 7]. In the same series of Nigam and colleagues, symptoms of leprosy preceded the symptoms of pulmonary TB and some patients had leprosy for a very long time (10–15 years) before TB [5]. In our series, three patients had leprosy on presentation and were found to have TB during investigations, two patients developed TB later while on treatment for leprosy, and one had a history of extrapulmonary TB 5 years before the diagnosis of leprosy. In the reported cases, the majority had pulmonary TB, whereas extrapulmonary TB was rare (TB of the larynx in a lepromatous patient [8], cutaneous TB [9], and central nervous system TB [3]). In our series, one patient developed extrapulmonary TB involving generalized lymphadenopathy while on treatment for leprosy. In the cases we report, a majority of our patients with pulmonary TB had positive Mantoux test, high ESR, radiological evidence of TB, and AFB in sputum. Nigam *et al*. also reported radiological evidence in 70% of cases and AFB in sputum in 80% of cases in their case series [5].

**Table 2:** Six cases of tuberculosis and leprosy coinfection

Patient	Age (years)	Sex	First infection	Leprosy spectrum	Presentation of tuberculosis	Time gap between two infections	Comorbidities
1	57	Female	Extrapulmonary tuberculosis	Borderline tuberculoid	Extrapulmonary tuberculosis later pulmonary tuberculosis	5 years	HIV
2	47	Female	Simultaneous detection of both conditions	Lepromatous leprosy	Pulmonary tuberculosis	Simultaneous	Diabetes
3	72	Male	Simultaneous detection of both conditions	Lepromatous leprosy	Pulmonary tuberculosis	Simultaneous	
4	59	Male	Simultaneous detection of both conditions	Borderline lepromatous leprosy	Pulmonary tuberculosis	Simultaneous	
5	54	Male	Leprosy	Borderline lepromatous leprosy	Pulmonary tuberculosis	6 months	
6	50	Male	Leprosy	Lepromatous leprosy	Extrapulmonary tuberculosis	12 months	Diabetes

*HIV* human immunodeficiency virus

Leprosy and TB coinfection in the same patient was first reported in 1954 [10] and later several cases were published in the literature. There is an apparent decline in the number of coinfections [3]. Different theories have been put forward to explain the interaction between leprosy and TB. Some investigators suggested cross-immunity between the two diseases leading to reduced susceptibility to TB in those who have acquired immunity against *M*. *leprae*. Chaussinand reported that the prevalence of leprosy and the prevalence of TB are inversely related to each other and that prior TB exposure can be protective against leprosy [11]. Lietman *et al*. also suggested that TB could have contributed to the decline of leprosy in Western Europe [12]. Ohara and collegues immunized mice with recombinant *Mycobacterium bovis* BCG and this has led to reduced multiplication of *M. leprae* in the foot pads of mice, which supports the theory of cross immunity [13]. However, Donoghue *et al*. argued that multibacillary leprosy has led to increased mortality from TB [14].

Patients immunocompromised due to HIV infection, diabetes mellitus, and immunosuppressive drugs are more susceptible to TB infection. Whether the use of steroids in the treatment of leprosy increases the development of TB is controversial. In this series, two patients were on prednisolone for leprosy when they developed TB. Sreeramareddy *et al.* [15], Prasad *et al*. [16], Trindade *et al*. [1], and Rawson *et al*. [3] also reported cases of patients who developed TB while they were on steroids for leprosy. Agarwal *et al*. [17] reported the case of a post renal transplant recipient on a low dose of steroids and azathioprine, who developed both TB and leprosy. Jick *et al*. concluded that patients treated with glucocorticoids have an increased risk of developing TB [18]. However, major trials using corticosteroids for prophylaxis and treatment of nerve function impairment in leprosy did not identify steroid use as a risk factor for TB [19, 20]. Patients with lepromatous leprosy have been demonstrated to have downregulated immune response due to lower tumor necrosis factor (TNF)-alpha and chemokine response [21], which can be a risk factor for the development of TB. Three of our patients had lepromatous leprosy, who developed TB. One had HIV with low CD4 count and another patient had diabetes as possible causes for immunosuppression for both infections.

Development of rifampicin resistance secondary to rifampicin monthly dosing in leprosy can be a major concern in coinfected patients. However, currently there are no case reports of rifampicin resistance identified in coinfected patients in the literature [3]. Our first patient was diagnosed to have rifampicin-resistant TB while she was on treatment for leprosy with rifampicin and she also had HIV infection and a past history of extrapulmonary TB. Kumar *et al.* stated the importance of recognizing the presence of TB in patients with leprosy to avoid monthly rifampicin to prevent the development of rifampicin-resistant TB [4].

## Conclusion

In this case series of leprosy and TB coinfection, a majority of our patients were men, had lepromatous leprosy, and developed leprosy first and a majority of our patients with pulmonary TB had positive Mantoux test, high ESR, radiological evidence, and AFB in sputum, which is compatible with previously reported cases in the literature. An immunocompromised status, such as HIV infection, diabetes, and immunosuppressive drugs, are risk factors for TB infection. The use of steroids in the treatment of leprosy may increase the susceptibility to develop TB. Development of rifampicin resistance secondary to monthly rifampicin in leprosy is a major concern in treating coinfected patients with TB. Despite the paucity of reports of coinfection, it is advisable to screen for TB in patients with leprosy, especially if there are respiratory or constitutional symptoms, high ESR, and abnormal CXR. Because positive Mantoux and QuantiFERON Gold tests and the presence of AFB in sputum are misleading, CXR evidence of active TB and positive TB cultures are important diagnostic clues for active TB infection in a patient with leprosy. This is important to avoid monthly rifampicin in patients with suspected coinfections, which may lead to development of drug resistance to TB treatment. Whether prolonged steroid therapy in leprosy is a risk factor for development of TB is still controversial.

## Data Availability

The data sets supporting the conclusions of this article are included within the article.

## Abbreviations

AFB: Acid-fast bacilli
BCG: Bacillus Calmette–Guérin
B/L: Bilateral
BMI: Body mass index
HIV: Human immunodeficiency virus
ESR: Erythrocyte sedimentation rate
CXR: Chest X-ray
TB: Tuberculosis
